# Impact of smoking exposure on meibomian gland morphology and tear film stability: a cross-sectional study

**DOI:** 10.3389/fmed.2025.1711567

**Published:** 2025-11-19

**Authors:** Ting Chen, Ruidong Wang, Shuxian Liang, Jingting Feng

**Affiliations:** 1 Department of Ophthalmology, Shanxi Cardiovascular Hospital, Taiyuan, Shanxi, China; 2 Department of Ophthalmology, First Hospital of Shanxi Medical University, Taiyuan, Shanxi, China

**Keywords:** meibomian gland, meibomian gland dysfunction, meibomian gland morphology, meibum, tear film break-up time, smoking index, oxidative stress, inflammation

## Abstract

**Purpose:**

To investigate the effects of smoking on the structure and function of the meibomian glands.

**Methods:**

A total of 104 chronic smokers (with a smoking history of more than 5 years) and 44 healthy non-smokers were included. Based on smoking index, participants were categorized into four groups: non-smoking, mild, moderate, and heavy smoking. Meibomian gland images were compared across groups to assess morphological features, including defect area, number, height, and width. Furthermore, eyelid margin morphology, meibum characteristics and quantity, as well as tear film breakup time (TBUT), were evaluated to further investigate the impact of smoking on the structure and function of the meibomian glands.

**Results:**

The gland dropout area demonstrated the strongest positive correlation with smoking index (*β* = 0.449, *p* < 0.001). The number, height, and width of meibomian glands were negatively correlated with smoking index (*β* = −0.258, *p* = 0.002; *β* = −0.192, *p* = 0.021; *β* = −1.176, *p* = 0.036, respectively). Furthermore, the eyelid margin morphology score and meibum secretion function worsened with increasing smoking index (*B* = 0.002, OR = 0.998, 95% CI: −0.001 to 0.003, *p* < 0.001, *B* = −0.002, OR = 0.998, 95% CI: −0.001 to −0.002, *p* < 0.001). Tear film stability showed negative correlations with smoking index (*β* = −0.245, *p* = 0.003).

**Conclusion:**

Increased smoking exposure is strongly associated with deleterious structural and functional alterations of meibomian glands. Increased smoking exposure is associated with more severe meibomian gland morphological damage, worsened eyelid margin signs, reduced meibum quality and quantity, and accelerated deterioration of tear film stability. The underlying mechanisms may involve oxidative stress and chronic inflammation induced by tobacco smoke. For the management of smoking-related meibomian gland dysfunction (MGD), smoking cessation should be advised along with targeted anti-inflammatory or antioxidant treatments.

## 1 Introduction

The Meibomian gland, situated within the tarsus, is oriented perpendicularly to the eyelid margin and features an opening at the margin through which meibum are excreted. This meibum forms the outermost layer of the tear film, contributing critically to its stability and protecting the ocular surface from pathogenic microorganisms and environmental allergens (1, 2). A reduction in the number of Meibomian glands, along with diminished quantity and quality of lipid secretion, leads to increased tear evaporation and tear film instability, ultimately resulting in Meibomian gland dysfunction (MGD) (3, 4). Clinical manifestations include dryness, aching, swelling, irritation, and inflammatory responses (5). Studies indicate that certain systemic conditions—such as Sjögren’s syndrome, rheumatoidarthritis, Stevens–Johnson Syndrome(SJS),and hypertension—are associated with an elevated risk of MGD (6).

In addition, established risk factors for MGD include aging, hormonal imbalances, orthokeratology lens wear, Demodex folliculorum infestation, and certain lifestyle factors (7–10). The detrimental effects of smoking extend beyond an increased risk of cancer and are closely associated with a variety of ocular diseases. Specifically, smoking has been linked to the development of glaucoma, age-related macular degeneration, cataract, optic neuritis, and thyroid-associated ophthalmopathy (11–13). Harmful constituents in smoke—including nicotine, nitrosamines, tar, hydrogen cyanide, polycyclic aromatic hydrocarbons, formaldehyde, carbon monoxide, as well as heavy metals and toxic mineral elements—exert a persistent irritant effect on the sensitive conjunctival mucosa. This irritation can manifest as ocular discomfort, such as stinging, burning, and epiphora (7–9). Furthermore, chronic exposure to smoke-filled environments not only elevates the risk of ocular diseases but may also contribute to pathological alterations of the ocular surface. Given that the pathogenesis of MGD involves multiple interacting factors, and smoking represents a clearly modifiable external factor with substantial evidence linking it to various ocular surface diseases, it is particularly necessary to control for and isolate the independent effect of such intervenable lifestyle factors in MGD research. By eliminating the interference of other confounding variables, we can more clearly elucidate the specific role of smoking in the development and progression of MGD, thereby providing a theoretical basis for targeted behavioral interventions and early prevention.

Previous studies exploring the link between smoking and MGD have led to varied results (Supplementary Table 1). A study by Wang et al. found that smokers with MGD had worse lid margin and meibum scores but, notably, no significant difference in tear film breakup time (TBUT) or ocular surface symptoms compared to non-smokers (5). Another work has demonstrated significant meibomian gland loss in chronic smokers (14). These discrepancies may arise from differences in study populations or measurement techniques, such as the use of fluorescein for TBUT assessment, which can itself induce reflex tearing and alter tear film instability. The present study was designed to clarify these findings by employing a quantitative, dose–response approach and utilizing non-invasive infrared imaging to assess a comprehensive range of structural and functional parameters in a cohort of smokers and non-smokers.

## 2 Materials and methods

### 2.1 Subjects

This prospective study recruited non-smoking and smoking subjects from the ophthalmology outpatient and consultation services of Shanxi Cardiovascular Hospital between January 2024 and December 2024. To minimize selection bias, participants were initially enrolled irrespective of the presence of ocular discomfort, such as dryness, burning, or tingling sensations. Subjects in the smoking group had no ocular pathology other than mild refractive errors. Exclusion criteria included a history of hordeolum, meibomian gland disease, keratoconjunctivitis (whether infectious or allergic in etiology), severe dry eye, pterygium, contact lens use, ocular surgery, or any ocular medication within the preceding 6 months. Additionally, individuals with systemic conditions such as Sjögren’s syndrome, Stevens–Johnson syndrome, rheumatoid arthritis, systemic lupus erythematosus, hyperthyroidism, or any other disease potentially affecting the ocular surface were excluded, as were those using medications for systemic disorders. Non-smoking participants were also free from ocular and systemic diseases, apart from mild refractive errors. They confirmed no personal history of smoking, no close familial or social contacts who smoked, and no occupational or residential exposure to secondhand smoke. There were no specific occupational restrictions for either group, with the exception of individuals employed in computer-based professions or aviation-related roles.

A total of 44 healthy non-smoking subjects (25 males, 19 females) and 104 smoking subjects (79 males, 25 females) were ultimately enrolled. All smokers had a chronic smoking history exceeding 5 years, with cigarette consumption ranging from 2 cigarettes per week to 3 packs per day. The mean smoking duration was 13.1 years (range: 5–45 years). The mean age of both groups was 60.252 ± 5.201 years. No statistically significant difference in age was observed between the two groups (*p* > 0.05). However, a significant difference in gender distribution was identified (*p* < 0.01).

This study was approved by the Institutional Review Board of Shanxi Cardiovascular Hospital (No. 2025xxg900) and was strictly conducted in accordance with the World Medical Association’s Declaration of Helsinki. Informed consent was obtained from all participants.

### 2.2 Method

All clinical examinations were performed by an experienced attending ophthalmologist who was blinded to the patients’ symptoms, lifestyle habits, and medical history. Related assessments were carried out using a dry eye imaging system manufactured by Chongqing Kanghua Ruiming Technology Co., Ltd. Evaluations of meibomian gland condition included: meibomian gland imaging analysis, eyelid margin imaging analysis, meibum analysis, and TBUT. To prevent potential bias, one eye was randomly selected from each participant for all measurements.

Procedure: The slit lamp was first activated to examine the patient’s eyes and exclude other ocular pathologies. The dry eye imaging system software was then launched on the computer.

#### 2.2.1 Meibomian gland imaging

Images were captured to evaluate the morphology of the meibomian glands, clearly identify areas of dropout, and perform quantitative analysis using the “Fiji” software (15–17).

The procedure was conducted as follows: engaging the dry eye imaging device → adjusting the magnification to 6x → selecting “Meibomian Gland Quantitative Analysis” in the software → automatically switching to infrared illumination with brightness set to maximum → positioning the illumination arm at 45° → elevating the upper eyelid to fully expose the tarsal plate → acquiring focused images → analyzing the results.

The following parameters were selected for statistical analysis: meibomian gland dropout ratio (calculated as 1 − [meibomian gland area/total tarsal area]), gland height, gland width, and number of glands.

#### 2.2.2 Eyelid margin analysis

Imaging was performed to evaluate the morphology of the Meibomian gland orifices, presence of lipid plugs, and overall condition of the eyelid margin.

The procedure was conducted as follows: the magnification was adjusted to 10 × → “Gland Orifice Observation” was selected in the software → the system automatically switched to visible light mode: the front illumination was turned off, while the background light was activated and adjusted to one- to two-thirds of maximum intensity → the eyelid margin was brought into clear focus and photographed → pathological changes were manually graded according to the following Reference Values:

- Grade 1: Orifices are clear and transparent.
- Grade 2: Mild hyperemia of the eyelid margin; lipid plugs may be present.
- Grade 3: Obtuse rounding and thickening of the eyelid margin, neovascularization, obstruction and elevation of Meibomian gland orifices, presence of lipid plugs, and/or fibrosis and atrophy of the openings.

#### 2.2.3 Meibum analysis

This examination utilizes specular reflection from the tear film to assess meibum diffusion. The resulting images reveal multiple areas on the cornea with deficient lipid distribution.

The procedure was conducted as follows: The “Meibum Analysis” module was selected within the software → this triggered an automatic switch to the visible light source, and the illumination intensity was adjusted to two-thirds of its maximum → the cornea was then brought into sharp focus, following which the slit-lamp was slightly pulled back to achieve an optimal view, Video recording was initiated once the dynamic movement and interference patterns of the meibum became clearly visible → based on a direct comparison of the observed meibum dynamics against standard reference patterns, the examiner manually assigned a meibum grade score for the patient.

Reference values:

- Grade 1: <15 nm
- Grade 2: ≈15 nm
- Grade 3: ≈30 nm
- Grade 4: ≈30–80 nm
- Grade 5: ≈80 nm
- Grade 6: ≈80–120 nm
- Grade 7: ≈120–160 nm.

#### 2.2.4 TBUT

TBUT was used to evaluate tear film stability, which serves as a primary.

clinical diagnostic criterion for dry eye. This imaging system eliminates the need for traditional topical fluorescein sodium application, thereby avoiding associated risks (2). The assessment allows for evaluation of the combined contribution of the major components of the precorneal tear film—aqueous, mucin, and lipid layers.

The procedure was conducted as follows: the magnification was adjusted to 16 × → “Tear Film Break-Up Time — Infrared Recording” was selected in the software → the cornea was brought into focus, and the patient was prompted to blink before recording commenced → the software automatically analyzed the TBUT value (a normal first TBUT is defined as >10 s).

Based on smoking status, subjects were categorized into four groups for analysis, including a non-smoking group. Smoking subjects were further classified into three subgroups according to the smoking index (number of smoking years × number of cigarettes smoked per day) (5): a heavy smoking group (smoking index ≥500), a moderate smoking group (smoking index 100–499), and a mild smoking group (smoking index <100). These were compared with the healthy non-smoking group.

### 2.3 Statistical analysis

Data for all continuous variables are presented as mean ± standard deviation. The normality of data distribution was assessed using the Anderson-Darling test. Given that age represents a known risk factor for meibomian gland dysfunction (MGD), study participants were selected within a narrow age range (60.25 ± 5.20 years). Multiple linear regression analyses were performed to control for potential confounding effects of gender while examining the independent influence of smoking index on meibomian gland parameters (dropout area, gland count, height, and width) and tear film break-up time. For ordinal outcome variables, including eyelid margin and meibum quality scores, ordered logistic regression was employed. A *p* < 0.05 was considered statistically significant. All statistical analyses were conducted using SPSS software (version 23).

## 3 Results

### 3.1 Meibomian gland morphological parameters

**Figure 1:**
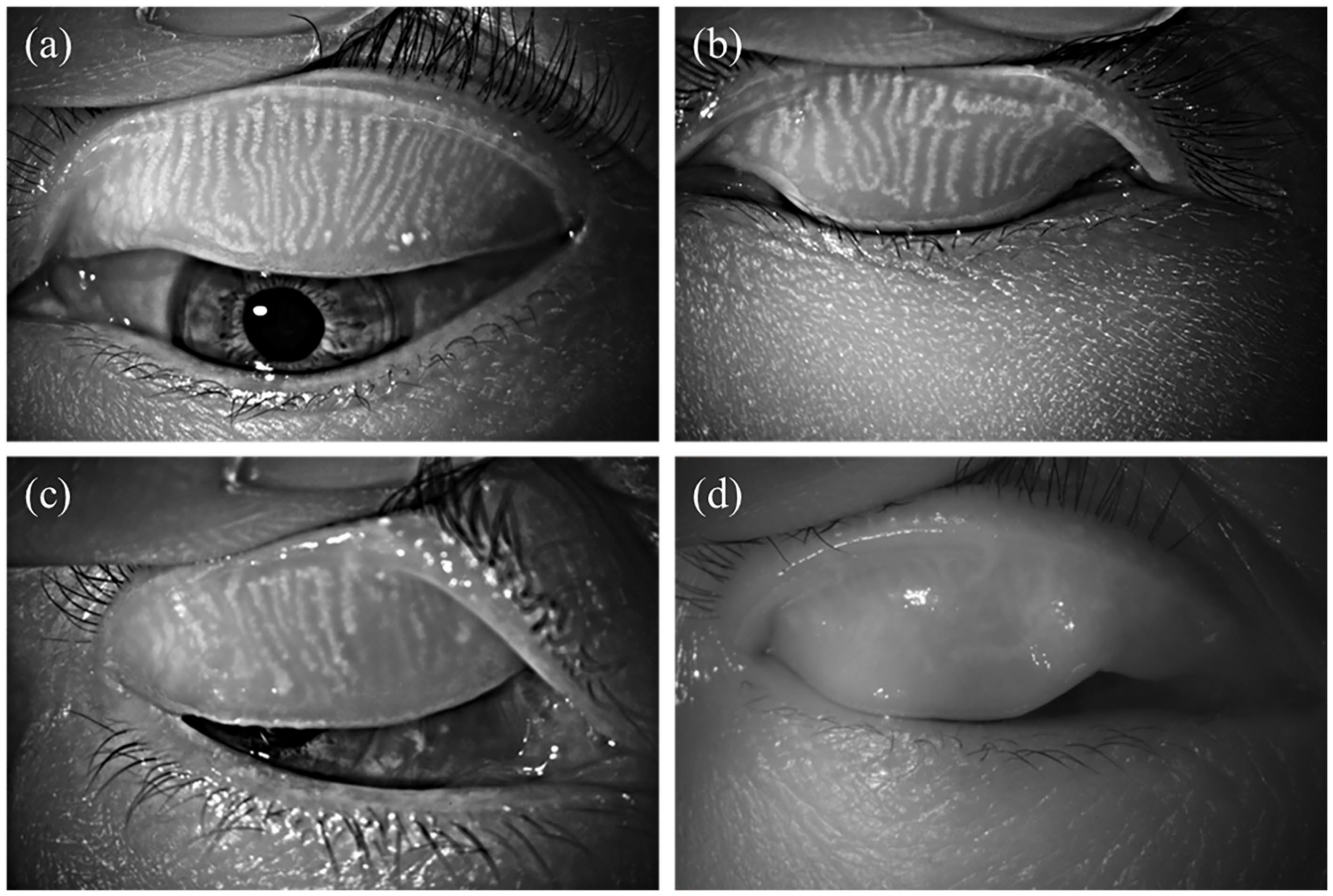
Representative meibography images of the upper eyelid in subjects with different smoking levels: **(a)** non-smoker; **(b)** mild smoker; **(c)** moderate smoker; **(d)** heavy smoker.

To investigate the effects of smoking on the meibomian glands, meibography was first performed to quantify the area of gland dropout. Related meibomian gland parameters were subsequently measured and analyzed. Representative original meibographs of the upper eyelids from subjects with varying smoking levels are presented in Figure 1. As shown in Figure 1a, the non-smoking group exhibited a meibomian gland dropout rate of approximately 29%. In the mild smoking group (Figure 1b), the dropout rate was about 45%. Subjects in the moderate smoking group (Figure 1c) showed a dropout rate of approximately 52%. Compared with the mild smoking group, these subjects demonstrated a slight increase in average gland width, reduced gland height, decreased number of glands, and localized dropout in the nasal region. The severe smoking group (Figure 1d) exhibited a meibomian gland dropout rate of approximately 68%, with extensive areas of gland loss, a reduced number of glands, as well as morphological alterations such as gland tortuosity and partial structural obscuration.

**Figure 2:**
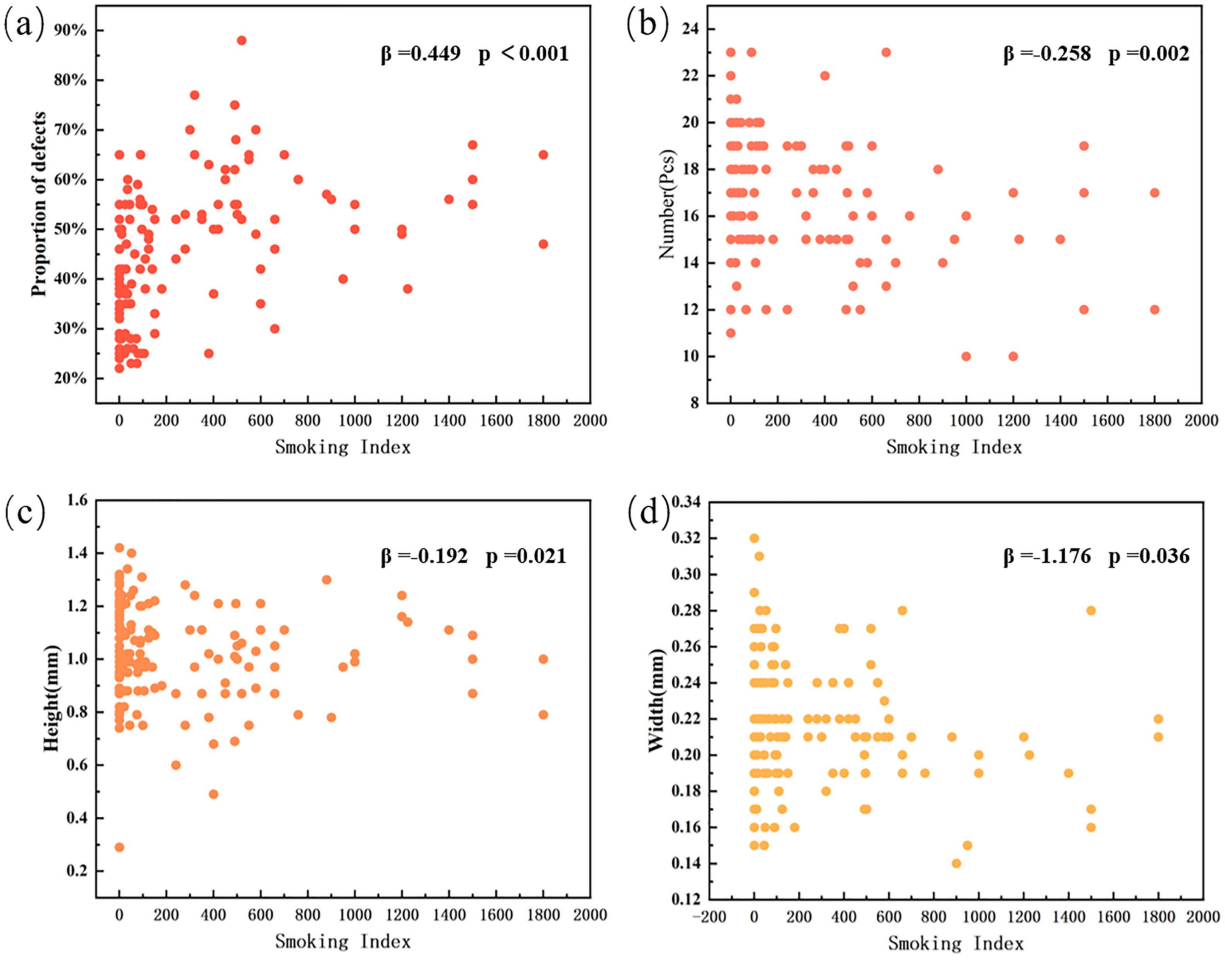
Smoking index and distribution of morphological parameters of meibomian gland. **(a)** Proportion of gland defects (β = 0.449, *p* < 0.001). **(b)** Number of meibomian glands (β = −0.258, *p* = 0.002). **(c)** Gland height (β = −0.192, *p* = 0.021). **(d)** Gland width (β = −1.176, *p* = 0.036).

Table 1 presents the meibomian gland measurement data of the upper eyelid in non-smokers and subjects with varying degrees of smoking exposure. Partial meibomian gland dropout was observed across all middle-aged and elderly subjects, though the extent of loss varied. Non-smokers exhibited the least glandular dropout, with a defect area of 0.348 ± 0.092. Their meibomian gland metrics included a count of 16.955 ± 2.753 glands, height of 1.048 ± 0.205 mm, and width of 0.219 ± 0.038 mm. As the level of smoking exposure increased, a progressive reduction was observed in the average number, height, and width of the meibomian glands, accompanied by increasing gland dropout. In heavy smokers, the gland count decreased to 15.276 ± 2.852, with height and width reduced to 1.007 ± 0.141 mm and 0.208 ± 0.034 mm. This group demonstrated the most severe meibomian gland loss, with a defect area of 0.542 ± 0.118, representing a 55.747% increase compared to non-smokers. Multivariate linear regression analysis was performed. Among the meibomian gland morphological parameters, the degree of meibomian gland loss showed a significant positive correlation with smoking index (*B* < 0.001, *β* = 0.449, *p* < 0.001), while the effect of gender was not statistically significant (*p* = 0.449). The number of meibomian glands (*B* = −0.002, *β* = −0.258, *p* = 0.002) was correlated with smoking index, with no statistically significant effect of gender (*p* = 0.620). Similarly, gland height (B < 0.001, *β* = −0.192, *p* = 0.021) demonstrated correlation with smoking index, and the gender effect remained statistically non-significant (*p* = 0.357). Gland width (B < 0.001, *β* = −1.176, *p* = 0.036) also showed correlation with smoking index, with no statistically significant gender effect (*p* = 0.485) (Figure 2). Specifically, meibomian gland number, height, and width decreased with increasing smoking index, whereas the extent of gland dropout increased. These results indicate that smoking is associated with marked morphological alterations in the meibomian glands, and that the severity of these structural changes escalates with higher levels of smoking exposure.

**Table 1:** Multivariate linear regression analysis of meibomian gland parameters with smoking (mean ± standard deviation).

Meibomian gland morphological parameters	Non-smoker	Mild smoker	Moderate smoker	Heavy smoker	β	*p*
*N*	44	41	34	29		
Proportion of defects	0.348 ± 0.092	0.402 ± 0.128	0.508 ± 0.130	0.542 ± 0.118	0.449	<0.001
Number	16.955 ± 2.753	17.122 ± 2.272	16.765 ± 2.583	15.276 ± 2.852	−0.258	0.002
Height (mm)	1.048 ± 0.205	1.055 ± 0.150	0.968 ± 0.194	1.007 ± 0.141	−0.192	0.021
Width (mm)	0.219 ± 0.038	0.226 ± 0.036	0.212 ± 0.026	0.208 ± 0.034	−0.176	0.036

### 3.2 Eyelid margin analysis

**Figure 3:**
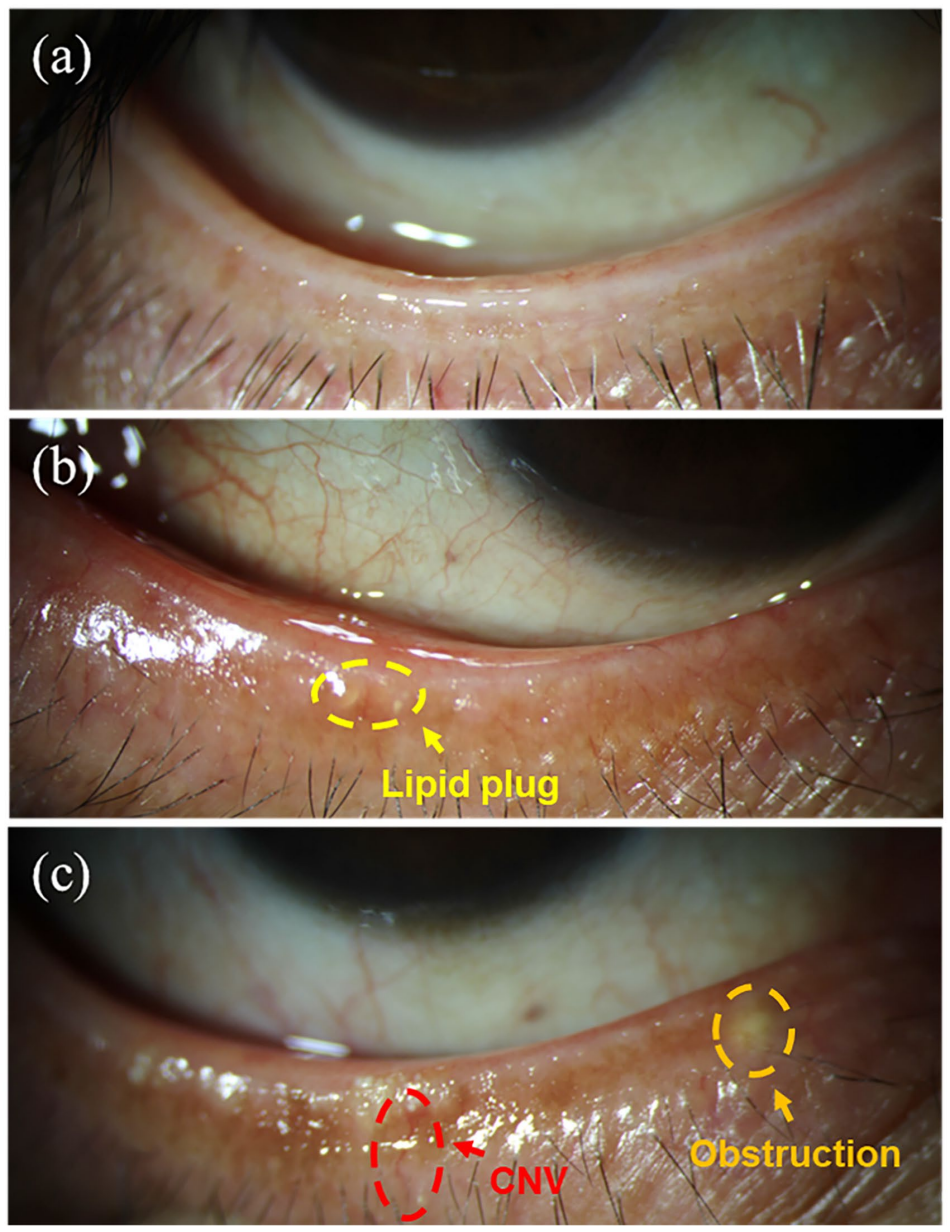
Typical images of the lower eyelid margin in subjects with different morphological grades: **(a)** Grade 1; **(b)** Grade 2; **(c)** Grade 3.

Relevant studies indicate that the condition of the eyelid margin, particularly the meibomian gland orifices, is an important factor in evaluating meibomian gland morphology (17). Therefore, we further examined the eyelid margins of subjects with different smoking intensities to investigate the impact of smoking on eyelid margin morphology. Figure 3 present representative clinical images of the lower eyelid margins from patients with different grades of morphological changes. Grade 1 morphology (Figure 3a) exhibits a normal eyelid margin without hyperemia or thickening. The meibomian gland orifices are patent, with clear and transparent meibum secretion. Grade 2 morphology (Figure 3b) shows mild hyperemia along the eyelid margin and a small number of meibum plugs. Grade 3 morphology (Figure 3c) is characterized by a rounded and thickened eyelid margin with neovascularization, The meibomian gland orifices appear obstructed and elevated, accompanied by numerous meibum plugs.

**Figure 4:**
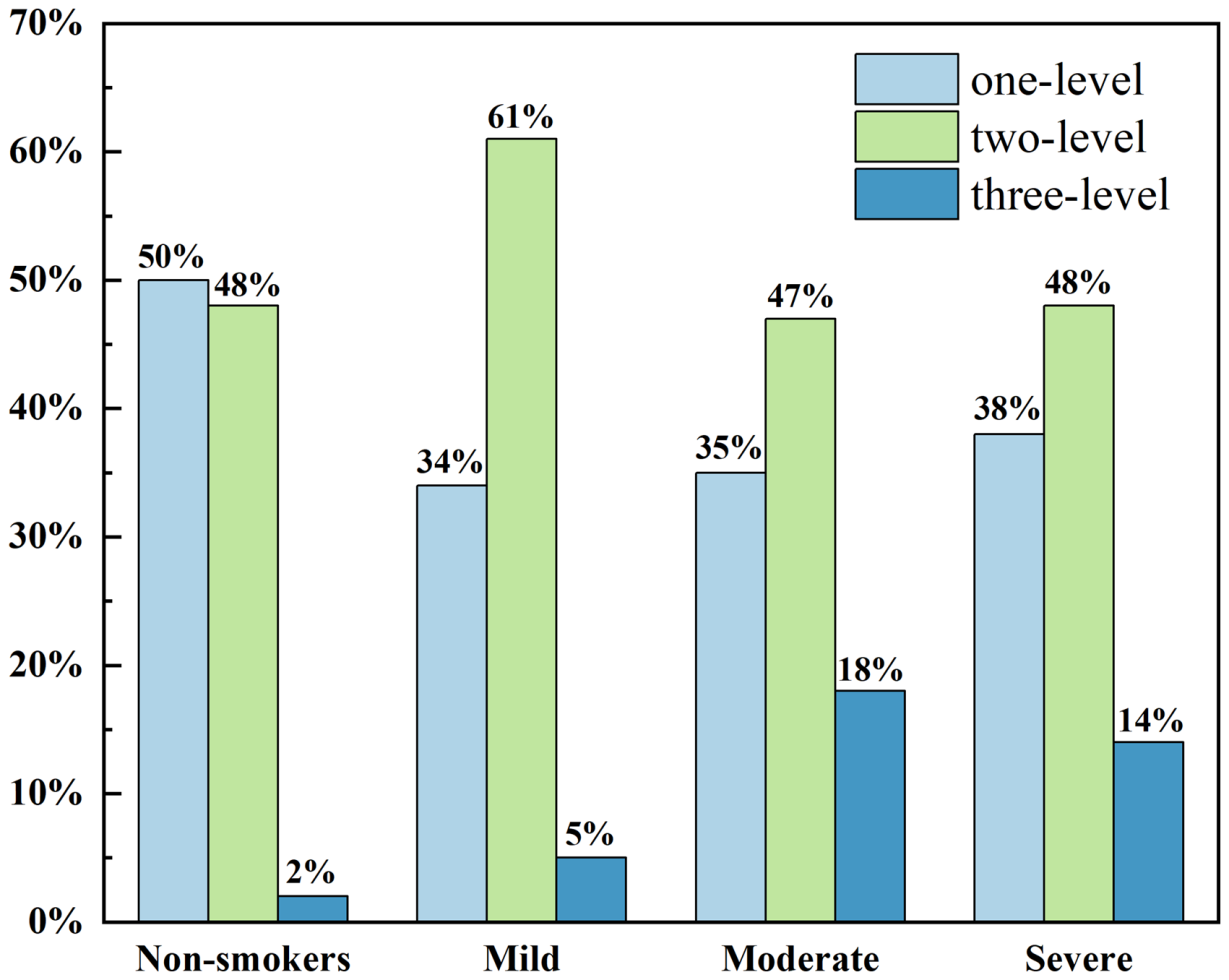
The distribution map of the lower eyelid margin grade value of subjects with different degrees of smoking.

Figure 4 shows the relationship between smoking intensity and the distribution of eyelid margin morphology grades. It is evident that non-smokers generally exhibit better eyelid margin morphology, with similar proportions of Grade 1 and Grade 2 morphology, while the proportion of Grade 3 morphology is extremely low. Among smokers, the proportion of Grade 1 morphology was generally lower than in non-smokers, while the proportion of Grade 3 morphology increased. In both moderate and heavy smoking groups, the distribution of the three eyelid margin morphology grades was relatively similar. The proportion of Grade 2 morphology in both groups was lower than in the light smoking group, while the proportion of Grade 3 morphology was significantly higher than in the light smoking group. Ordered logistic regression analysis was performed to evaluate the dose–response relationship between smoking index and ordinal eyelid margin morphology scores. The analysis identified smoking index as a significant predictor for deterioration in eyelid margin grading (*B* = 0.002, OR = 0.998, 95% CI: −0.001 to 0.003, *p* < 0.001). In contrast, the association between gender and eyelid margin morphology scores was not statistically significant (*p* = 0.871). These results confirm that long-term cumulative smoking exposure significantly increases the risk of morphological deterioration of the eyelid margin.

### 3.3 Meibum analysis

**Figure 5:**
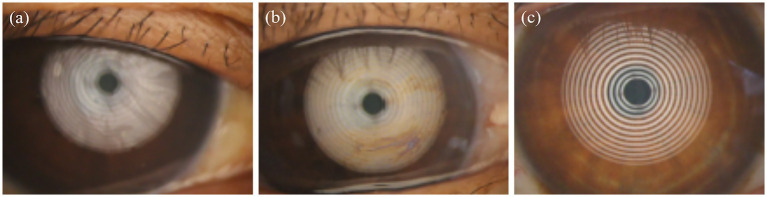
Lipid diffusion patterns in subjects with different smoking intensities: **(a)** Grade 7; **(b)** Grade 4; **(c)** Grade 1.

To further investigate the impact of smoking on meibomian gland function beyond morphological abnormalities, we evaluated the quality and quantity of secreted meibum by observing their diffusion patterns. Meibum quality was graded on a seven-point scale, where higher grades indicated better secretion function. Representative images of meibum diffusion patterns across different grades are presented in Figure 5. Figure 5a illustrates a Grade 7 meibum diffusion pattern, characterized by abundant ocular surface meibum that spread smoothly and completely across the ocular surface after blinking. As meibum secretion function declines, Figure 5b shows a Grade 4 pattern, where meibum diffusion is observed only in certain areas after blinking, followed by early breakup, uneven meibum distribution, and localized absence of meibum over the temporal cornea. In contrast, a Grade 1 diffusion pattern in Figure 5c demonstrates nearly absent meibum diffusion on the ocular surface.

**Figure 6:**
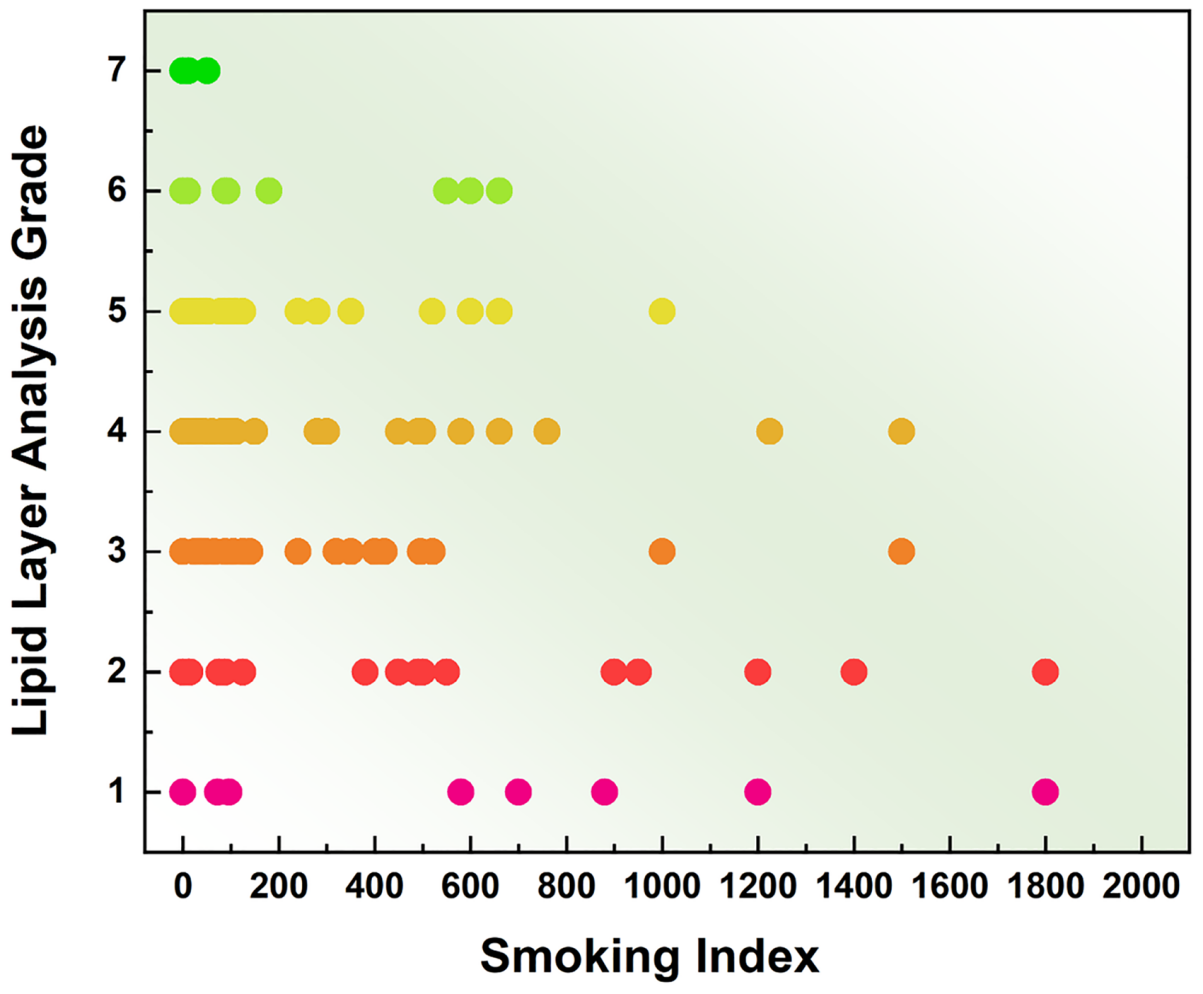
Distribution of mean meibum grades among subjects with different smoking intensities.

Non-smoking subjects exhibited lipid layer grades predominantly ranging from Grade 3 to Grade 6. The majority showed smooth meibum diffusion with abundant meibum secretion. In contrast, smokers generally had lower meibum diffusion grades, which were concentrated between Grade 2 and Grade 6. Specifically, the mild smoking group primarily displayed grades between 3 and 5, the moderate group between 2 and 5, and the heavy smoking group between 1 and 4. The distribution of average meibum layer grades across the four groups is presented in Figure 6. Ordered logistic regression analysis was conducted to evaluate the dose–response relationship between smoking index and ordinal meibum diffusion grade scores. Smoking index was identified as a significant predictor for deterioration in meibum diffusion grading (*B* = −0.002, OR = 0.998, 95% CI: −0.001 to −0.002, *p* < 0.001). In contrast, the association between gender and meibum grade showed no statistical significance (*p* = 0.810). These results confirm that smoking significantly impairs the secretory function of meibomian glands, leading to reduced quantity and quality of meibum secretion.

### 3.4 TBUT

**Figure 7:**
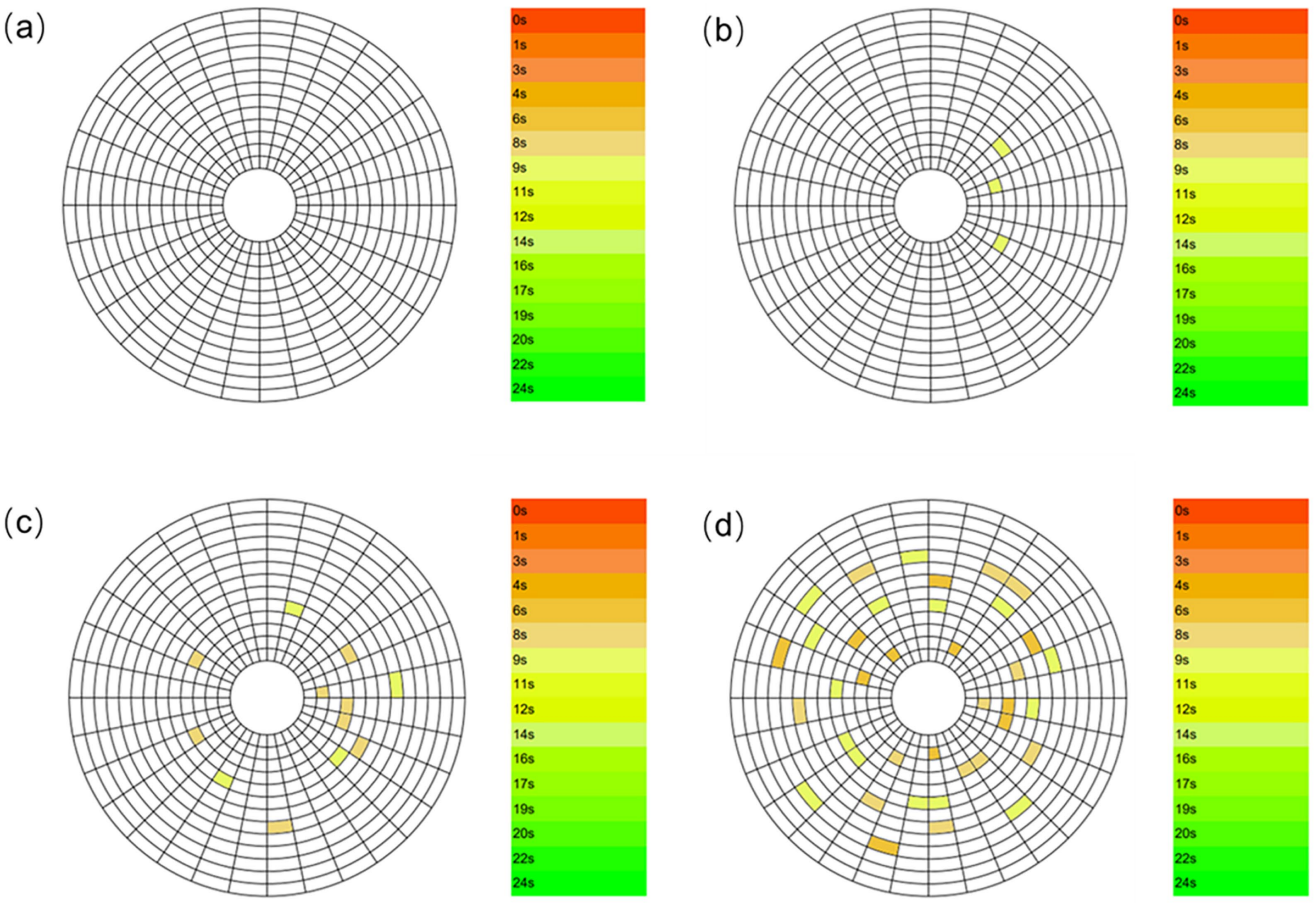
TBUT patterns in subjects with different smoking levels: **(a)** Non-smoker; **(b)** mild smoker; **(c)** moderate smoker; **(d)** heavy smoker.

Meibum constitutes an essential component of the tear film and serves to inhibit excessive tear evaporation. A normal TBUT typically exceeds 10 s; values below this threshold indicate tear film instability. Therefore, measurement of TBUT is an important indirect method for evaluating meibomian gland function (2). Figure 7 illustrates schematic representations of TBUT patterns in subjects with different levels of smoking exposure. Non-smoking subjects (Figure 7a) exhibited no tear film break-up within 10 s, indicating a stable tear film. The first break-up time (fBUT) and average break-up time (avBUT) in this group were 10.591 ± 3.699 s and 12.341 ± 3.882 s, respectively. Notably, the large standard deviation suggests variable tear film stability even among non-smokers. Figure 7b presents the TBUT pattern in mild smokers, who displayed relatively few break-up points within 10 s. Their fBUT and avBUT decreased to 9.537 ± 3.828 s and 10.585 ± 3.578 s. With increased smoking exposure, moderate smokers (Figure 7c) revealed more corneal break-up points within 10 s compared to the mild group. Their fBUT and avBUT were further reduced to 8.059 ± 3.756 s and 8.734 ± 3.467 s. Heavy smokers (Figure 7d) demonstrated extensive corneal break-up within 10 s. This group had the shortest break-up times, with an fBUT of 6.448 ± 4.145 s and an avBUT of 7.862 ± 4.077 s.

**Figure 8:**
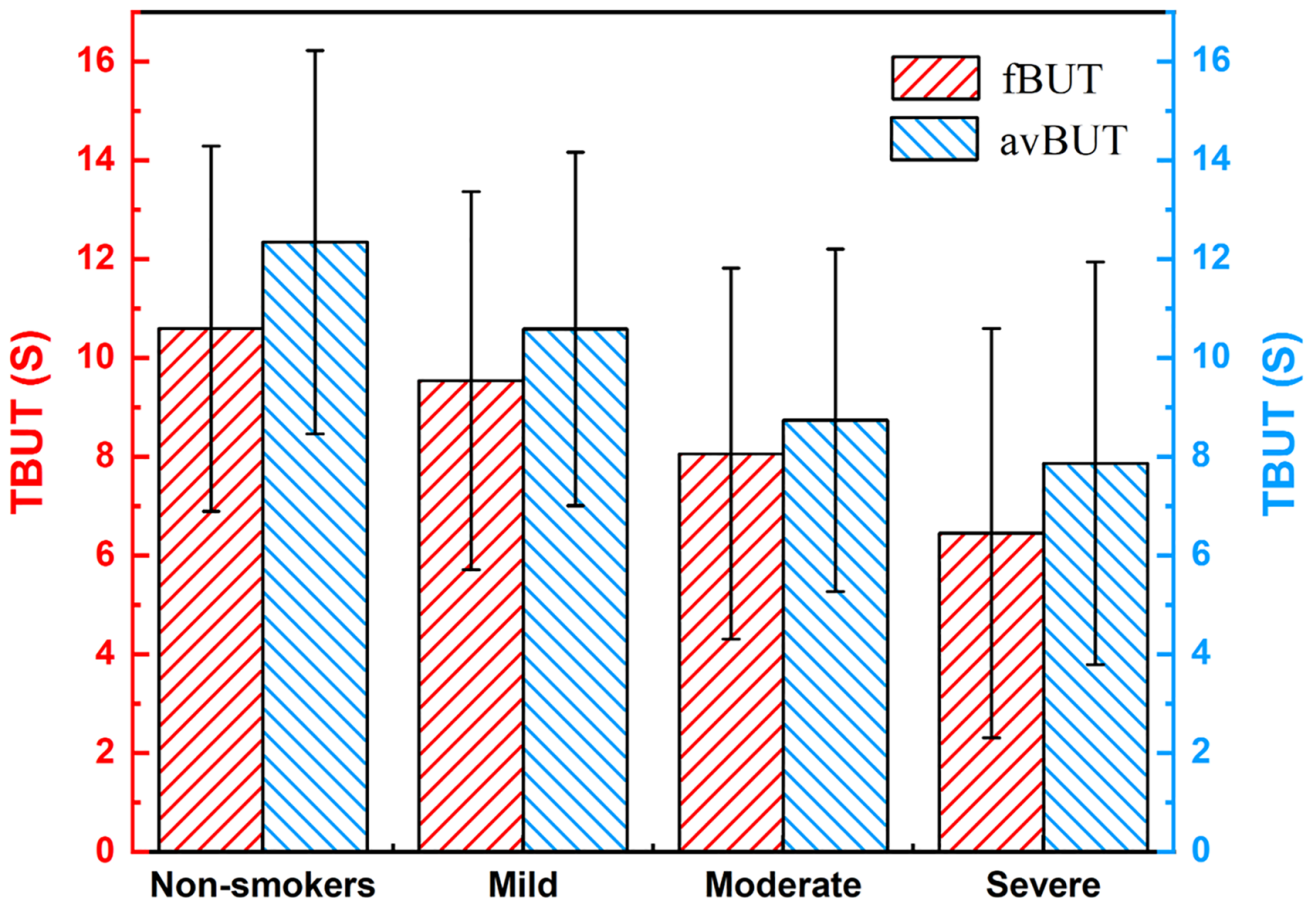
TBUT in subjects with different smoking levels.

Figure 8 presents the TBUT among subjects with varying levels of smoking exposure. Both the fBUT and the avBUT exhibited a gradual decrease as smoking intensity increased. Multiple linear regression analysis revealed a significant negative correlation between smoking index and fBUT (*B* = −0.003, *β* = −0.245, *p* = 0.003), with no statistically significant effect of gender (*p* = 0.238). Similarly, smoking index showed a significant negative correlation with avBUT (*B* = −0.03, *β* = −0.260, *p* = 0.002), and the effect of gender again remained non-significant (*p* = 0.194). These results indicate that smoking exposure serves as an independent risk factor for decreased tear film stability.

## 4 Discussion

In clinical practice, we have observed that a considerable number of patients with structural and functional impairments of the meibomian glands have a history of smoking. Those with longer smoking duration and higher daily cigarette consumption are more likely to present marked clinical symptoms such as ocular dryness, distending pain, soreness, and general discomfort. In severe cases, these may be accompanied by ocular pain and photophobia.

The present study further confirms that the smoking index is correlated with multiple objective parameters, including meibomian gland morphology, eyelid margin morphology scores, meibum quality, and TBUT. With increasing smoking index, patients demonstrate expanded meibomian gland dropout area, reduced gland height, width, and number, more severe eyelid margin abnormalities, greater susceptibility to gland orifice obstruction, as well as decreased meibum secretion function and compromised tear film stability.

Previous studies by Wang et al. reported that, compared to non-smokers, smokers exhibit various abnormal eyelid margin signs, including irregularity, telangiectasia, meibomian gland orifice obstruction, and anterior or posterior displacement of the mucocutaneous junction. In addition, meibum quality deteriorated, becoming increasingly turbid and viscous with higher smoking index. It is noteworthy that their study found no significant effect of smoking on TBUT (5). Consistent with this, Muhafiz et al. also concluded that smoking affects meibomian gland morphology but not TBUT (14).

The discrepancies between the findings of this study and previous reports may be attributed to differences in the methodology used for measuring TBUT. Earlier studies commonly employed an invasive approach, which involves instilling sodium fluorescein into the eye and subsequently evaluating it under blue light using a slit-lamp microscope with a yellow enhancement filter (18–21). However, the dye itself may stimulate reflex tearing, thereby disrupting tear film stability and influencing the measurement outcomes (22, 23). In contrast, this study utilized a non-invasive measurement technique based on the principle of Placido disk projection. By analyzing tear film reflection images, this method allows for assessment over a broader corneal area, up to 10 mm in diameter (24). Since this approach avoids chemical interference with the tear film, the obtained non-invasive TBUT values are generally lower than those acquired via the fluorescein method, with reported mean differences of up to 3.7 s (25, 26), this discrepancy decreases as the baseline TBUT value decreases. It is noteworthy that Srivastav et al., also using a non-invasive approach, confirmed a significant correlation between meibomian gland dropout and shorter NIBUT values in a healthy population. This finding aligns with the results of the present study: smoking concurrently leads to more severe meibomian gland dropout and lower NIBUT values, collectively revealing the dual detrimental effects of smoking on both the structure of the meibomian glands and the stability of the tear film (27).

In summary, smoking can lead to significant structural and functional alterations in the meibomian glands, particularly impairing the natural function of the lipid layer. However, the precise mechanisms underlying smoking-induced changes in meibomian gland structure and function have not yet been fully elucidated and are likely to involve multiple pathways.

Tobacco smoke comprises approximately 4,000 active compounds, the vast majority of which demonstrate toxicity following acute or chronic exposure (4, 28). Key toxic constituents include carbon monoxide, methanol, aldehydes, nitrosamines, hydrocarbons, and heavy metals (17). Previous research indicates that tobacco smoke impairs the structure and function of the meibomian glands primarily via oxidative stress and inflammatory pathways.

### 4.1 Oxidative stress

Tobacco smoke contains reactive oxygen and nitrogen species that can induce oxidativestress (29, 30). Studies have shown that each puff of cigarette smoke contains approximately 10^14^ free radicals in the tar phase and about 10^15^ in the gas phase, including both long-lived semiquinones and short-lived reactive oxygen species (2, 17). Smokers inhale substantial quantities of potentially harmful free radicals from tobacco, resulting in significant free radical exposure. Reactive oxygen species act as potent oxidants that attack biomolecules through chain reactions (28, 31). At physiological concentrations, these species participate in regulating various cellular processes, including apoptosis, inflammation, innate immunity, wound healing, and the activity of transcription factors and their surface receptors (18). However, excessive intracellular production of reactive oxygen species can cause oxidative damage to DNA, proteins, lipids, and metabolites, potentially leading to aberrant gene and protein expression, protein aggregation, and cellular dysfunction (31–33). Chronic oxidative stress may overwhelm endogenous antioxidant defenses, resulting in cell necrosis, tissue dysfunction, and DNA mutations. Furthermore, cigarette smoke inhalation rapidly depletes circulatory antioxidant reserves (29), thereby exacerbating lipid peroxidation and sustaining a state of persistent oxidative damage (34).

Tobacco smoke induces oxidative damage via reactive oxygen species, leading to the generation of peroxidation products including lipid peroxides (LPO), hexanoyl-lysine (HEL), 4-hydroxy-2-nonenal (4-HNE), malondialdehyde (MDA), and other endogenous toxic aldehydes and their derivatives (35–37). Among these, HEL originates from oxidative modification of *ω*-6 fatty acids such as linoleic acid and arachidonic acid, serving as a reliable biomarker for early-stage peroxidation. Acrolein, one of the most irritating components in tobacco, functions both as a tobacco combustion product and an endogenous metabolite generated during lipid peroxidation (11, 14, 38, 39). The study by Rummenie et al. demonstrated that tear levels of HEL and acrolein increase significantly within 5 min to 24 h following smoke exposure, confirming that tobacco smoke rapidly induces intense oxidative stress in ocular tissues (13). Furthermore, these key lipid peroxidation biomarkers remain elevated in the tear film and ocular surface tissues of chronic smokers, indicating persistent oxidative stress in the eye. Lipid molecules exhibit particular susceptibility to oxidative stress and readily undergo lipid peroxidation when attacked by reactive oxygen species (28, 40). Consequently, during ocular oxidative stress, meibomian gland lipids become vulnerable to oxidative damage, resulting in impaired meibum secretion. As patients’ smoking index increases, sustained oxidative stress progressively alters the physical characteristics of meibum. The secreted lipid transitions from a transparent liquid to a viscous, paste-like consistency (41, 42). Since meibum constitutes a crucial component of the tear film, these physicochemical changes directly compromise tear film stability, ultimately leading to shortened tear film break-up time. In summary, tobacco smoke impairs the structural integrity and physiological function of the tear film lipid layer through oxidative stress and lipid peroxidation pathways. Even brief or passive exposure to cigarette smoke can induce ocular surface tissue injury, with more pronounced effects observed in long-term smokers (13). However, the precise relationship between smoking exposure and structural alterations in the meibomian glands requires further investigation (5).

### 4.2 Inflammatory response

Smoking induces persistent low-grade chronic oxidative damage in ocular tissues, with injury severity progressing over exposure duration. This sustained, low-level oxidative stress maintains the immune system in a state of continuous mild activation to preserve homeostasis, resulting in para-inflammation (2, 17, 43). While para-inflammation serves as an adaptive mechanism to maintain or restore tissue integrity and function in the short term, prolonged para-inflammatory states promote inflammatory cell accumulation and may ultimately lead to fibrosis. Thus, para-inflammation represents a controlled, transient, and protective physiological response under normal conditions. However, persistent smoking exposure disrupts this regulatory balance, facilitating the transition to chronic inflammation. Furthermore, clinical studies have confirmed that reactive oxygen species activate the NLRP3 inflammasome through the ROS-NLRP3-IL-1β signaling pathway, thereby promoting inflammation (44, 45). Cigarette smoke exposure enhances oxidative stress in epithelial cells, and the resulting accumulation of reactive oxygen species activates inflammatory signaling pathways that initiate inflammatory responses (39, 46). Concurrently, elevated inflammation further amplifies oxidative stress through a positive feedback mechanism, sustaining both inflammatory activity and oxidative damage at persistently high levels in ocular tissues.

Smoking modulates the release and inhibition of both pro-inflammatory and anti-inflammatory mediators. Cigarette smoke stimulates kinases such as ERK and JNK, along with transcription factors including NF-κB, thereby enhancing the expression of pro-inflammatory cytokines such as TNF-*α*, TNF-α receptors, interleukin (IL)-1, IL-6, IL-8, and granulocyte-macrophage colony-stimulating factor (GM-CSF) (31, 47). A study by Shin KK and colleagues examining the IL-6-572C > G polymorphism in healthy Korean males demonstrated that carriers of this variant exhibited an augmented inflammatory response to smoking over time (11, 48). Conversely, smoking has also been associated with reduced IL-6 production via Toll-like receptors (TLR)-2 and 9, decreased IL-10 production following TLR-2 activation, and diminished secretion of IL-1β, IL-2, TNF-α, and IFN-*γ* in monocytes (39, 49, 50).

Inflammatory mediators, including both pro-inflammatory and anti-inflammatory factors (39, 49), directly suppress gene expression in conjunctival goblet cells and induce their apoptosis (51). Concurrently, these factors activate epithelial keratinization pathways, promoting squamous metaplasia and the consequent loss of normal physiological function. These pathological changes exacerbate tissue damage and further stimulate inflammatory cell infiltration, establishing a self-perpetuating cycle of pathology (7, 18, 52). The meibomian gland orifice, located at the eyelid margin, represents a transitional zone between the keratinized stratified squamous epithelium of the skin and the non-keratinized stratified columnar epithelium of the conjunctiva, which contains abundant goblet cells. This junctional region is particularly susceptible to squamous metaplasia under inflammatory conditions, representing a key pathological feature in meibomian gland dysfunction. Rummenie and colleagues performed impression cytology before and 24 h after smoke exposure to directly evaluate conjunctival goblet cell density and the extent of squamous metaplasia. Healthy conjunctival epithelial sheets demonstrated abundant goblet cells, whereas 24 h post-exposure, a marked reduction in goblet cell density was observed, accompanied by pronounced squamous metaplasia and inflammatory cell infiltration. Immunohistochemical staining for MUC5AC in impression cytology specimens revealed numerous MUC5AC-positive goblet cells prior to cigarette smoke exposure. In contrast, a significant decrease in MUC5AC-positive cells was documented 24 h after exposure. Concurrently, tear IL-6 concentrations increased substantially following smoke exposure, confirming that smoking elevates inflammatory mediator levels on the ocular surface (13, 53). Furthermore, meibum quality correlates closely with leukocyte infiltration within meibomian glands. Increased CD45^+^ cells in both acini and ducts associate with reduced lipid secretion and altered composition (29, 54, 55). Chavance and colleagues further demonstrated that CD45^+^ cell numbers rise significantly with increasing smoking intensity (56). Collectively, these findings indicate that tobacco smoke promotes chronic inflammation and immune cell infiltration, ultimately leading to structural and functional alterations in meibomian glands (57, 58).

Although individuals with significant second-hand smoke exposure were excluded from this study, other environmental factors—such as ambient air pollution (e.g., PM2.5) or occupational pollutant exposure—were not quantitatively assessed. These unmeasured variables represent potential residual confounding factors. Future studies incorporating personal exposure monitoring would be valuable to disentangle the specific effects of cigarette smoke from those of other environmental pollutants.

In summary, smoking induces both oxidative stress and chronic inflammation, resulting in structural and functional alterations of the meibomian glands. Current management of MGD primarily involves local symptomatic treatments—such as artificial tears, gland massage, and warm compresses—which often provide only short-term symptomatic relief (11, 59). Based on the results of this study, the use of antioxidant and lipid-based eye drops may represent a more sustainable therapeutic approach by directly counteracting oxidative damage to the meibum caused by free radicals derived from smoking. Furthermore, a critical objective in MGD treatment is to control or even reverse squamous metaplasia induced by chronic inflammation. Effective management should include elimination of irritants, smoking cessation, avoidance of secondhand smoke, and judicious use of anti-inflammatory agents such as antibiotic or corticosteroid ointments to mitigate inflammation. At the same time, the management of smoking-related MGD should extend beyond smoking cessation recommendations to include comprehensive environmental controls (60). Our findings position the ocular surface and meibomian glands as sensitive sentinel windows for monitoring inhalable insults beyond tobacco smoke. Beyond tobacco smoke, gasses from wildfire smoke, household biomass fuel combustion, urban traffic emissions, industrial pollutants, and e-cigarette aerosols also contain reactive aldehydes, particulate matter, and reactive oxygen species. Therefore, investigating the impact of these increasingly prevalent environmental and occupational exposures on meibomian gland dysfunction represents a critical future research direction, fostering interdisciplinary integration between ophthalmology and public environmental health.

## Data Availability

The raw data supporting the conclusions of this article will be made available by the authors, without undue reservation.
