# Supplementary material for: Impact of smoking exposure on meibomian gland morphology and tear film stability: a cross-sectional study
Source: Front Med (Lausanne). 2025 Nov 19;12:1711567. doi: 10.3389/fmed.2025.1711567 (PMC12672861; doi:10.3389/fmed.2025.1711567)
Supplement: Supplementary file 1 [file Table_1.docx]

Supplemental Table 1 Summary of TBUT values for active smokers and non-smokers.

|  | Method | Smokers | Non-smokers | Significant  difference |
| --- | --- | --- | --- | --- |
| Wang et al.(2016) [1] | sodium fluorescein | 3.96±0.05 | 4.00±0.14 | No |
| Muhafiz et al.(2019) [2] | sodium fluorescein | 11.23±5.94 | 9.65±6.14 | No |
| Ağın et al.(2020) [3] | sodium fluorescein | 10.52±2.25 | 10.96±3.64 | No |
| Carreira et al.(2023) [4] | sodium fluorescein | 5.40±1.72 | 12.23±1.34 | Yes(p<0.001) |
| Kalayci et al.(2023) [5] | sodium fluorescein | 9.84±2.13 | 6.96±2.31 | Yes(p=0.002) |
| This study | non-invasive approach | 8.19±4.05 | 10.59±3.7 | Yes(p=0.003) |

[1] S. Wang, H. Zhao, C. Huang, Z. Li, W. Li, X. Zhang, and Z. Liu, Impact of Chronic Smoking on Meibomian Gland Dysfunction, PLoS ONE 11, e0168763 (2016).

[2] E. Muhafiz, S. Aslan Bayhan, H. A. Bayhan, and C. Gürdal, Effects of chronic smoking on the meibomian glands, Int Ophthalmol 39, 2905 (2019).

[3] A. Ağın, S. Kocabeyoğlu, D. Çolak, and M. İrkeç, Ocular Surface, Meibomian Gland Alterations, and In Vivo Confocal Microscopy Characteristics of Corneas in Chronic Cigarette Smokers, Graefes Arch Clin Exp Ophthalmol 258, 835 (2020).

[4] A. R. Carreira, S. Rodrigues-Barros, J. C. Silva, M. F. De Almeida, I. Machado, J. N. Cardoso, and N. Campos, Tobacco effects on ocular surface, meibomian glands, and corneal epithelium and the benefits of treatment with a lipid-based lubricant, Graefes Arch Clin Exp Ophthalmol 261, 171 (2023).

[5] M. Kalayci, E. Cetinkaya, L. Yaprak, K. Yigit, E. Suren, B. Dogan, and M. K. Erol, Ocular surface assessment and morphological alterations in meibomian glands with non-contact meibography in electronic cigarette smokers, ABO 86, (2023).
